# Supplementary material for: Astragalus Polysaccharide Modulates the Gut Microbiota and Metabolites of Patients with Type 2 Diabetes in an In Vitro Fermentation Model
Source: Nutrients. 2024 May 30;16(11):1698. doi: 10.3390/nu16111698 (PMC11174380; doi:10.3390/nu16111698)
Supplement: Supplementary file 1 [file nutrients-16-01698-s001.zip › nutrients-2989336-supplementary.pdf]

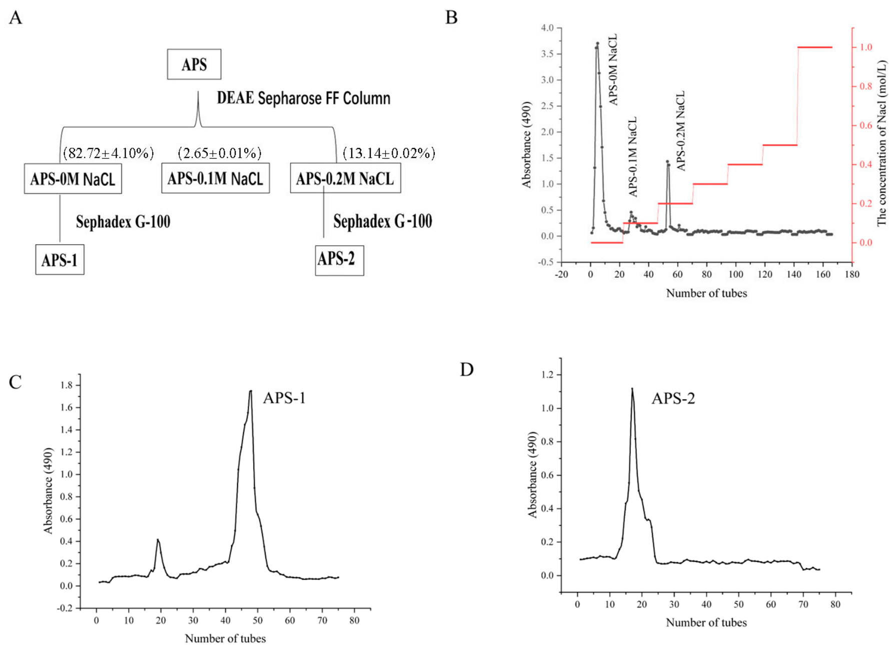


Supplementary Figure S1 Purification of APS. (A) Purification procedure of APS. (B) Stepwise elution curve of APS on DEAE Sepharose FF chromatography column. Elution curve of APS-1(C) and APS-2 (D) on Sephadex G-100 chromatography column.

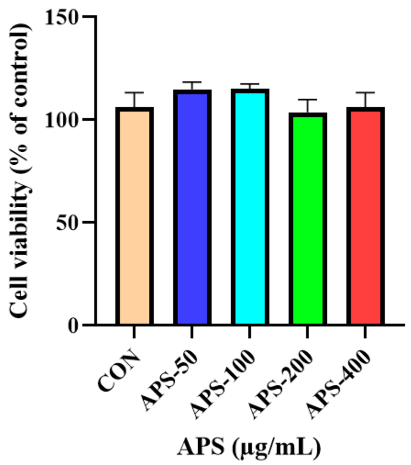


Supplementary Figure S2. The effects of APS on the viability of Caco-2 cells for 24 h in different concentrations (50, 100, 200, 400 μg/mL). Cell viability was determined using the MTT assay.

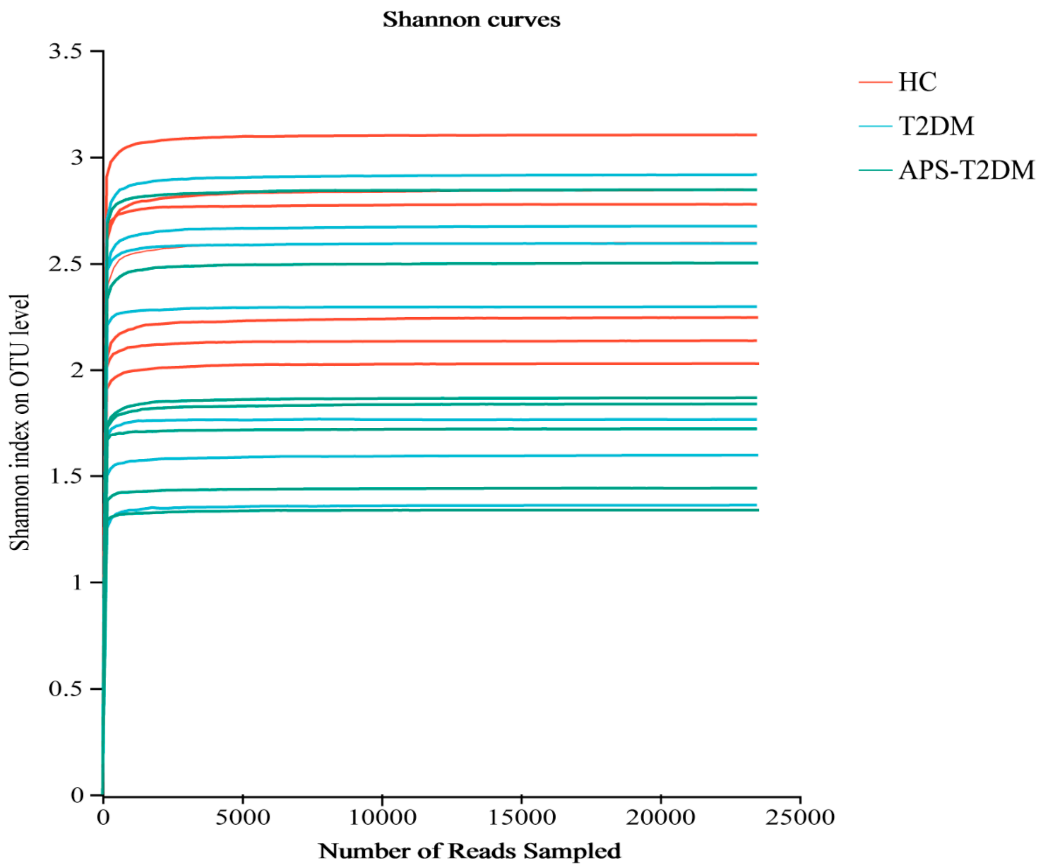


Supplementary Figure S3 Dilution curve

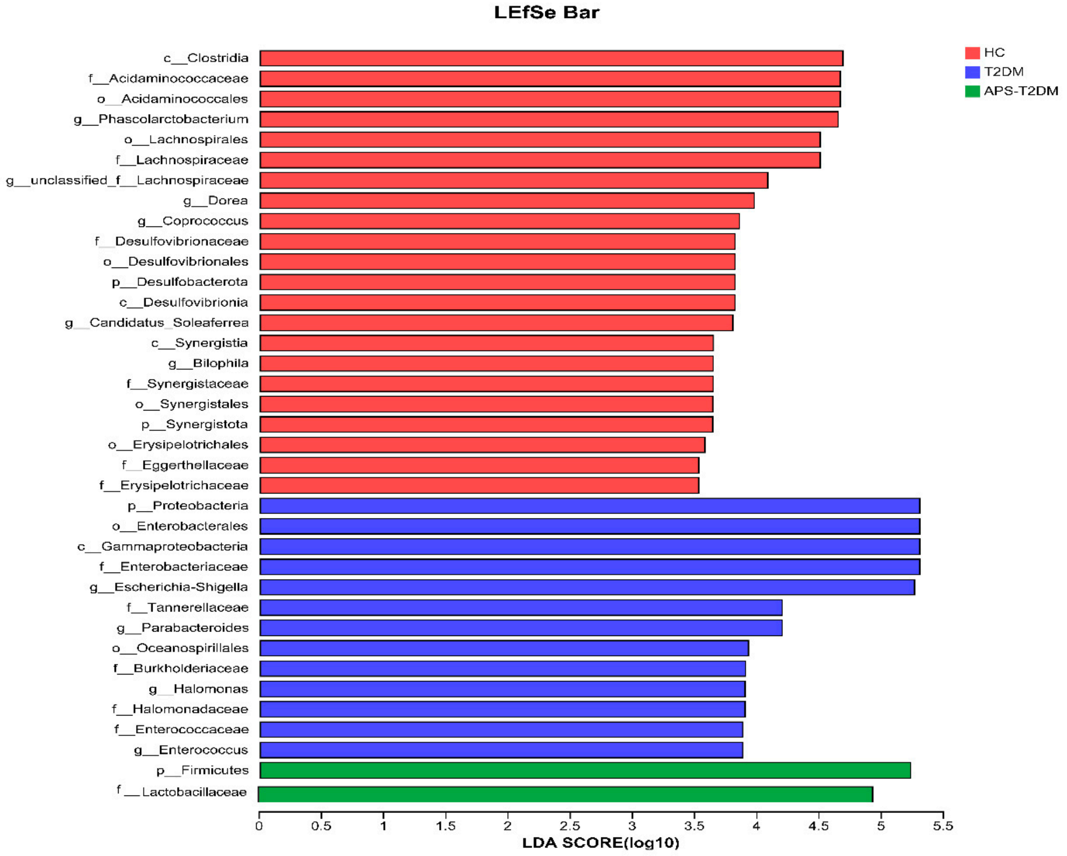


Supplementary Figure S4. Linear discriminant analysis (LDA) score f gut microbial taxa among groups

Supplementary Table S1 Preparation of simulated digestion solution stock solution

| | SGF（mmol/L） | SIF（mmol/L） |
|---|---|---|
| KCl | 6.9 | 6.8 |
| $KH_2PO_4$ | 0.9 | 0.8 |
| NaHCO3 | 25 | 85 |
| NaCl | 47.2 | 38.4 |
| $MgCl_2(H_2O)_6$ | 0.1 | 0.33 |
| $(NH_4)_2CO_3$ | 0.5 | —— |

Supplementary Table S2 Basic physicohemical properties and structure analysis of APS

| **Chemical composition (%, g/g)** | **APS** | **APS-1** | **APS-2** |
|---|---|---|---|
| Extractive yield of crude polysaccharides | 9.48±0.54 | 59.92±0.70 | 32.21±0.20 |
| Carbohydrates | 75.73±0.72 | 95.71±1.42 | 92.50±1.25 |
| Proteins | 0.079±0.002 | - | - |
| Total phenol | - | - | - |
| **Monosaccharide composition (molar, %)** | | | |
| Arabinose | 1 | - | 1 |
| Galactose | 0.33 | 1 | 0.85 |
| Glucose | 4.36 | 9.25 | 0.96 |
| Galacturonic acid | 0.02 | - | 0.46 |
| **Mw (KDa)** | | | |
| | 1928.54, 396.31, 239.92 and 4.87 | 4.39 | 1915.15 |

Supplementary Table S3 Metabolites of fermentation samples

| Compound | NC | | T2DM | | APS-T2DM | |
|---|---|---|---|---|---|---|
| | Average | SD | Average | SD | Average | SD |
| Methylamine | 7100898 | 3190710 | 6410459 | 2146446 | 3951929 | 2024370 |
| L-Alanine | 6719944 | 10760765 | 18579976 | 12539251 | 11415511 | 12706581 |
| 2-Aminobutanoic acid | 426665.7 | 552152.3 | 1061266 | 835449.2 | 343155.7 | 490293.3 |
| 1,3-Propanediol | 3597887 | 1340048 | 3310754 | 1289355 | 1727786 | 943267.8 |
| n-Butyric acid tetrahydrofurfuryl ester | 10576.27 | 15018.49 | 7940.683 | 9260.081 | 0 | 0 |
| Lactic Acid | 1585149 | 923426.1 | 4372554 | 5446577 | 32395404 | 35976566 |
| Glycolic acid | 328578.8 | 131176.9 | 333117.1 | 145703.5 | 230475.3 | 103589.1 |
| L-Valine | 14629669 | 12910063 | 25504991 | 12387776 | 10363767 | 10027640 |
| 2-Hydroxybutyric acid | 416562.6 | 376046.7 | 1077385 | 1295075 | 270599.9 | 240068.5 |
| Hydracrylic acid | 151585.1 | 137011.1 | 251269.8 | 259077.1 | 775682.8 | 1109743 |
| 2-Hydroxy-3-methylbutyric acid | 104431.3 | 168394.7 | 77467.45 | 189755.7 | 0 | 0 |
| L-Proline | 7294969 | 5571419 | 14061278 | 9051360 | 5422940 | 3415790 |
| 2-Hydroxyisocaproic acid | 379972.1 | 331109.5 | 500055.7 | 364173.2 | 248221.2 | 159034.3 |
| L-Serine | 714570.6 | 507611.6 | 1338245 | 717176.4 | 2336851 | 2200321 |
| L-Leucine | 15720485 | 14018599 | 31127449 | 12024880 | 19766231 | 12506779 |
| Niacin | 35812.85 | 37510.11 | 113480.1 | 75277.8 | 54632.92 | 44648.49 |
| L-Isoleucine | 7141492 | 7228488 | 13253276 | 7621969 | 5955610 | 4930428 |
| Glycine | 883962.9 | 897543.3 | 5812493 | 3943735 | 5315935 | 5531211 |
| Butanedioic acid | 905420.2 | 985001.3 | 2212689 | 3230161 | 5596897 | 11802410 |
| 2,3-Dihydroxy-2-methylpropanoic acid | 463956.6 | 193730.6 | 547885.9 | 259094 | 325654 | 169668.4 |
| Uracil | 618644.4 | 620104.3 | 1303425 | 871961 | 388623.1 | 318740.9 |
| Amphetamine | 447455.9 | 413472.3 | 461795.1 | 199365.3 | 1441212 | 2088750 |

| | | | | | | |
|---|---|---|---|---|---|---|
| Serine | 361839 | 426245.3 | 909547.3 | 563592.4 | 842643.2 | 952715.5 |
| 2,3-Butanediol | 82366.13 | 100774.5 | 184121.8 | 136046.1 | 97838.4 | 88608.73 |
| L-Threonine | 817820.9 | 623823.2 | 2310848 | 1203124 | 21180.91 | 51882.42 |
| Pentanedioic acid | 73191.56 | 71747.66 | 3403.06 | 8335.761 | 5092.036 | 12472.89 |
| L-Methionine | 1352008 | 1100295 | 2233953 | 2036864 | 860029.7 | 464807.6 |
| Aspartic acid | 508665.3 | 310881.9 | 559629.9 | 475443.1 | 1100325 | 985916.5 |
| Tryptamine | 595933.2 | 465260.1 | 312018.7 | 399573.1 | 0 | 0 |
| L-5-Oxoproline | 9220316 | 9105930 | 18966117 | 17748451 | 18244911 | 12508114 |
| 4-Aminobutanoic acid | 402881.6 | 415412.1 | 1609671 | 1915739 | 13995662 | 16396218 |
| L-Cysteine | 33274.82 | 81506.32 | 62562.43 | 92213.43 | 2352631 | 1815493 |
| Phenethylamine | 197303.2 | 233672.1 | 59536.13 | 75292.96 | 0 | 0 |
| 3-Phenyllactic acid | 180272.4 | 134115 | 144505.8 | 160441 | 143985.3 | 150153.8 |
| Cadaverine | 11622856 | 7970804 | 4005964 | 3672247 | 810550.3 | 716049.6 |
| L-Glutamic acid | 658918.6 | 845205.7 | 6749477 | 5949507 | 1217635 | 1566031 |
| 5-Aminovaleric acid | 14408672 | 10027922 | 4444205 | 9133105 | 327378.5 | 801910.2 |
| Phenylalanine | 7086046 | 10510741 | 11024911 | 8510549 | 10663048 | 10863730 |
| Homocysteine | 8808.044 | 21575.21 | 10911.92 | 26728.64 | 0 | 0 |
| DL-Ornithine | 307538 | 332156.1 | 1372988 | 1763858 | 1540854 | 1806945 |
| Phloretic acid | 7449.279 | 11787.72 | 13573.36 | 15780.47 | 3334.535 | 8167.908 |
| L-Ornithine | 420929.5 | 669095.5 | 4763717 | 5891467 | 12467241 | 15840724 |
| Tyrosine | 1687433 | 1150589 | 2824119 | 4176694 | 774623 | 892456.5 |
| Tyramine | 3717395 | 3638384 | 2784043 | 3085517 | 473720.8 | 564408 |
| L-Lysine | 716352.9 | 833018.7 | 1112141 | 1171831 | 2191979 | 2479765 |
| L-Tyrosine | 1341580 | 580220.5 | 106192 | 65988.16 | 531367 | 747485.2 |
| Dopamine | 542211.5 | 447074.8 | 93030.82 | 99657.27 | 224147.5 | 434484.4 |
| Palmitic Acid | 2919096 | 1640119 | 2300124 | 1088117 | 2232206 | 1620670 |

| | | | | | | |
|---|---|---|---|---|---|---|
| 1-Octadecanol | 41579.59 | 29150.74 | 33303.98 | 16742.09 | 38260.17 | 37349.49 |
| 2,3-Butanedione | 155144.8 | 249381.5 | 161969.9 | 396743.7 | 28778.94 | 70493.72 |
| Stearic acid | 2096340 | 1678097 | 1415607 | 1388851 | 1319452 | 915774.5 |
| L-Tryptophan | 1457562 | 1975008 | 3682813 | 2776187 | 4014142 | 4751041 |
| Homoserine | 55270.7 | 135385 | 17410.46 | 42646.73 | 2815167 | 2684979 |
| Pipecolic acid | 430338.4 | 290728 | 876657 | 846860.4 | 3106005 | 6090586 |
| 1-Monopalmitin | 699415.4 | 345119.7 | 551880.5 | 335366.3 | 638301.2 | 374196.1 |
| 2-Butenedioic acid | 22556.59 | 36530.96 | 38081.91 | 49191.47 | 38861.86 | 51544.53 |
| 9H-Purin-6-ol | 373514.5 | 312175.4 | 604476 | 589017.5 | 84025.27 | 205819 |
| D-Mannitol | 64091.02 | 120909.3 | 2431.966 | 5957.076 | 441245.4 | 675923 |
| D-Sorbitol | 0 | 0 | 0 | 0 | 0 | 0 |
| Myo-Inositol | 5547.222 | 13587.86 | 297061.9 | 548424.7 | 1183866 | 1023602 |
| Scyllo-Inositol | 5080.817 | 8069.725 | 10413.38 | 14065.76 | 0 | 0 |
| Spermine | 30865.33 | 36682.31 | 47191.68 | 115595.5 | 73297.4 | 88431.1 |
| Sucrose | 0 | 0 | 0 | 0 | 239856.1 | 311464.1 |
| Adenine | 82008.18 | 82386.32 | 69744.05 | 85777.69 | 142810.1 | 174995.5 |
| Benzenepropanoic acid | 61907.58 | 95945.78 | 22770.49 | 55776.09 | 0 | 0 |
| Malic acid | 84748.54 | 101681.5 | 288611 | 273467.3 | 113705.1 | 226055 |
| Pyroglutamic acid | 400839 | 457200.5 | 2429556 | 2378343 | 455389.1 | 988260.8 |
| Citric acid | 12972.23 | 31775.34 | 104133 | 212836.4 | 3218214 | 5660573 |
| Pantothenic acid | 0 | 0 | 31851.82 | 53335.5 | 0 | 0 |
| Ethanolamine | 110409.9 | 141608.4 | 257081.1 | 214996.7 | 195633.2 | 266794.3 |
| Glycerol | 31842.86 | 77998.75 | 6807.563 | 16675.05 | 6437008 | 15767384 |
| 5-Hydroxytryptophan | 0 | 0 | 0 | 0 | 150202.5 | 180235.8 |
| Tricarballylic acid | 9452.946 | 23154.89 | 0 | 0 | 172616.4 | 149224.9 |
| Putrescine | 9179877 | 10756207 | 8126689 | 9541879 | 2193175 | 2922442 |

| | | | | | | |
|---|---|---|---|---|---|---|
| Xanthine | 43650.64 | 106921.8 | 0 | 0 | 109423.1 | 190602.3 |
| L-Cystine | 0 | 0 | 0 | 0 | 5952354 | 9443077 |
| 3-Hydroxyflavone | 0 | 0 | 0 | 0 | 0 | 0 |
| 2-Pyrrolidinone | 0 | 0 | 42620.85 | 67954.81 | 267843.4 | 425836.7 |
| Oxalic acid | 501304.4 | 1227940 | 881950 | 2160328 | 2379012 | 5718100 |
| Acetic acid ethenyl ester | 23350.85 | 57197.67 | 239257.2 | 342215.9 | 8140833 | 19940888 |
| D-Gluconic acid | 0 | 0 | 0 | 0 | 858329.4 | 479204.9 |
| 2-(Dimethylamino)ethanol | 0 | 0 | 76551.38 | 187511.8 | 354740.9 | 546205.1 |
| 2-Hydroxy-2-methylbutyric acid | 70520.85 | 129047.9 | 92403.55 | 109586.1 | 0 | 0 |
| Acetic acid | 21.6989 | 2.519824 | 13.69641 | 3.038647 | 18.1182 | 2.881895 |
| Propanoic acid | 7.577614 | 1.403171 | 5.7636 | 0.642027 | 16.9491 | 6.375782 |
| Butanoic acid | 5.164457 | 0.974737 | 1.798743 | 0.984722 | 4.001457 | 1.571232 |
| Isobutyric acid | 0.315114 | 0.198959 | 0.179299 | 0.13999 | 0.300886 | 0.084063 |
| Valeric acid | 0.087486 | 0.06069 | 0.0462 | 0.057687 | 0.085024 | 0.060946 |
| Isovaleric acid | 0.285961 | 0.094066 | 0.172871 | 0.046947 | 0.115914 | 0.073496 |
| Vitamin K1 | 1507437 | 438723.6 | 861689.5 | 498034.5 | 1089410 | 359349.6 |
| Uridine | 28611.97 | 3643.392 | 16768.72 | 11733.59 | 24471.05 | 5683.549 |
| Tryptophan | 1315571 | 949412.4 | 1700337 | 562049.1 | 2340486 | 173097.5 |
| Thymine | 95636.26 | 130003.9 | 19836.36 | 31576.58 | 0 | 0 |
| Thiamine | 254966.6 | 190700.3 | 448948.8 | 205096.2 | 1030596 | 255107.6 |
| Spermidine | 39909.15 | 19924 | 26377.58 | 13965.75 | 0 | 0 |
| Serotonin | 17806.85 | 36555.9 | 5977.398 | 14641.57 | 27627.03 | 36431.88 |
| Inosine | 1051442 | 944205.8 | 1418544 | 760638 | 1032041 | 615919.1 |
| Histidine | 131307.6 | 72367.98 | 129181.6 | 136780.7 | 145776.6 | 110218.7 |
| Histamine | 246147.5 | 117431.3 | 193715.3 | 139963.4 | 235296.5 | 31657.45 |
| Harman | 549255.2 | 214882 | 377425.9 | 152846.9 | 182295 | 67794.09 |

| | | | | | | |
|---|---|---|---|---|---|---|
| Glutamine | 27579.55 | 22582.51 | 72157.37 | 73691.3 | 228698.8 | 48111.24 |
| Biotin | 93219.59 | 40137.59 | 28286.98 | 37104.84 | 31285.26 | 36635.99 |
| Acetylcholine | 19868.48 | 12648.37 | 28048.61 | 14658.85 | 40455.13 | 5479.566 |
| 5,6-Dihydrouracil | 918362.6 | 628073.7 | 1034206 | 326975.6 | 1472369 | 62389.15 |
| 3-Formylindole | 3805953 | 2518275 | 4572155 | 2395094 | 7984612 | 299334.2 |
| sn-Glycero-3-phosphocholine | 250954.1 | 195095.9 | 68576.78 | 13768.76 | 94684.24 | 64326.87 |
| Pyridoxamine | 102299.1 | 21957.16 | 96760.42 | 6026.591 | 98067.36 | 14308.96 |
| L-Carnosine | 28137.77 | 48831.03 | 69258.11 | 80751.33 | 160987.3 | 66096.03 |
| Guanosine | 0 | 0 | 0 | 0 | 113844.2 | 195767.5 |
| Guanine | 78867.58 | 57799.8 | 47104.59 | 78356.35 | 192574.3 | 94120.83 |
| Glycocyamine | 2170200 | 1314010 | 2691753 | 1245321 | 4395122 | 175741.7 |
| all-trans-Retinoic acid | 28296.93 | 69313.04 | 95733.39 | 82127.06 | 581695.1 | 205036.5 |

Supplementary Table S4 Screening and identification of differential metabolites in different treatment groups

| Compound | T2DM vs HC | | | APS-T2DM vs T2DM | | |
|---|---|---|---|---|---|---|
| | Fold change | *P* value | VIP | Fold change | *P* value | VIP |
| L-Tyrosine | 0.080 | 0.000 | 2.273 | 5.004 | 0.847 | 0.935 |
| Pentanedioic acid | 0.100 | 0.039 | 1.441 | 1.496 | 0.917 | 0.190 |
| L-Glutamic acid | 9.832 | 0.029 | 1.570 | 0.184 | 0.049 | 1.254 |
| Glycine | 6.576 | 0.011 | 1.709 | 0.915 | 0.749 | 0.094 |
| Dopamine | 0.175 | 0.033 | 1.448 | 2.409 | 0.642 | 0.436 |
| sn-Glycero-3-phosphocholine | 0.273 | 0.041 | 1.612 | 1.381 | 0.482 | 0.675 |
| Niacin | 3.107 | 0.042 | 1.519 | 0.481 | 0.125 | 0.993 |
| Butanoic acid | 0.348 | 0.000 | 2.272 | 2.225 | 0.013 | 1.500 |
| L-Threonine | 2.826 | 0.019 | 1.695 | 0.020 | 0.001 | 1.853 |

| | | | | | | |
|---|---|---|---|---|---|---|
| Biotin | 0.354 | 0.016 | 1.826 | 0.354 | 0.016 | 1.826 |
| Homoserine | 0.315 | 0.917 | 0.513 | 73.497 | 0.026 | 1.344 |
| L-Cysteine | 1.880 | 0.298 | 0.408 | 34.113 | 0.009 | 1.511 |
| D-Gluconic acid | / | / | / | 15.515 | 0.001 | 1.756 |
| Tricarballylic acid | 0.000 | 0.317 | 0.810 | 8.195 | 0.019 | 1.410 |
| Spermidine | 0.661 | 0.125 | 1.120 | 0.154 | 0.001 | 1.787 |
| all-trans-retinoic acid | 3.383 | 0.077 | 1.003 | 5.860 | 0.000 | 1.934 |
| Guanine | 0.597 | 0.206 | 0.698 | 3.712 | 0.014 | 1.492 |
| Uracil | 2.107 | 0.140 | 1.144 | 0.299 | 0.032 | 1.312 |
| Propanoic acid | 0.761 | 0.002 | 1.766 | 2.941 | 0.001 | 1.774 |
| Glutamine | 2.616 | 0.396 | 1.022 | 2.921 | 0.001 | 1.826 |
| L-Proline | 1.928 | 0.142 | 1.206 | 0.386 | 0.049 | 1.226 |
| L-Valine | 1.743 | 0.277 | 1.061 | 0.406 | 0.038 | 1.272 |
| Thiamine | 1.761 | 0.142 | 1.086 | 2.296 | 0.001 | 1.774 |
| Harman | 0.687 | 0.085 | 1.106 | 0.483 | 0.014 | 1.501 |
| 9H-Purin-6-ol | 1.618 | 0.561 | 0.654 | 0.228 | 0.040 | 1.144 |
| Myo-Inositol | 53.551 | 0.063 | 0.883 | 3.985 | 0.025 | 1.063 |
| L-Carnosine | 2.461 | 0.333 | 0.800 | 2.324 | 0.022 | 1.270 |

The screening conditions for differential metabolites were VIP> 1, $P < 0.05$, FC > 2 or FC < 0.5. "/"Indicated that the substance was not detected
